# Evaluation of contact-active antibacterial properties of cetylpyridinium chloride–graphene oxide coatings on dental restorative and titanium surfaces: *an in vitro* study

**DOI:** 10.1186/s12903-026-07986-4

**Published:** 2026-02-24

**Authors:** Keisuke Okubo, Gen Kano, Masato Komoda, Hideyuki Kamata, Shin Nakamura, Yuki Shinoda-Ito, Kazuhiro Omori, Yuta Nishina, Shogo Takashiba

**Affiliations:** 1 Department of Periodontics and Endodontics, Field of Medical Development, Okayama University, 2-5-1 Shikata-cho, Kita-ku, Okayama, 700-8525 Japan; 2 Graduate School of Medicine, Dentistry and Pharmaceutical Sciences, Okayama University, 2-5-1 Shikata-cho, Kita-ku, Okayama, Okayama 700-8525 Japan; 3 Research Institute for Interdisciplinary Science, Okayama University, Tsushima-naka, Kita-ku, Okayama, 700-8530 Japan; 4 Department of Pathophysiology - Periodontal Science, Faculty of Medicine, Dentistry and Pharmaceutical Sciences, Okayama University, 2-5-1 Shikata-cho, Kita-ku, Okayama, 700-8525 Japan

**Keywords:** Wash-resistant antibacterial coating, Graphene oxide, Cetylpyridinium chloride, Oral pathogenic bacteria

## Abstract

**Objective:**

Biofilm formation on dental restorative materials and implant surfaces plays a central role in the development of dental caries, periodontal disease, and peri-implantitis. Durable antimicrobial surface treatments that inhibit bacterial adhesion and biofilm formation remain a significant unmet need in restorative and implant dentistry. Therefore, this study aimed to develop a composite coating combining cetylpyridinium chloride and graphene oxide, and to evaluate its durable antibacterial surface modification under in vitro conditions.

**Methods:**

A composite coating consisting of cetylpyridinium chloride and graphene oxide was prepared and applied to composite resin and titanium surfaces. Antibacterial activity against *Streptococcus mutans* and *Porphyromonas gingivalis* was evaluated using adenosine triphosphate assays and fluorescence-based live/dead staining. Coating retention after washing and air-drying was assessed by optical microscopy and Raman spectroscopy.

**Results:**

Cetylpyridinium chloride-graphene oxide-coated surfaces showed a significant reduction in bacterial viability compared with phosphate-buffered saline, ethanol, and cetylpyridinium chloride-only controls. Antibacterial effects were maintained after rinsing and air-drying on both composite resin and titanium surfaces. Raman spectroscopy confirmed the persistence of characteristic graphene oxide bands after washing, indicating stable retention of the coating on the material surfaces.

**Conclusions:**

Cetylpyridinium chloride–graphene oxide coatings demonstrate sustained surface-associated antibacterial activity against key cariogenic and periodontal pathogens and remain stably adhered to common dental restorative and implant materials after washing. These findings suggest that cetylpyridinium chloride–graphene oxide coatings may serve as a durable contact-active surface modification strategy to reduce biofilm formation associated with dental caries and peri-implantitis.

## Background

Dental caries and periodontal disease remain among the most common chronic infectious diseases worldwide and are leading causes of tooth loss and declining oral health [1, 2]. Both dental caries and periodontal disease are initiated by the adhesion and accumulation of multispecies biofilms on tooth surfaces and restorative materials [3, 4]. These biofilms contribute to enamel demineralization in dental caries and to the destruction of periodontal tissues in periodontal disease. Once established, these biofilms exhibit strong resistance to standard antimicrobial treatments, making their prevention and control a key focus of oral health research [5, 6]. Notably, peri-implantitis shares similar microbial causes and disease pathways with periodontal disease, including the involvement of anaerobic bacteria such as *Porphyromonas gingivalis* (*P. gingivalis*) [7, 8]. As the use of dental implants increases worldwide, these overlapping biofilm-related infections have become a serious clinical concern, emphasizing the urgent need for preventive strategies effective for both natural teeth and dental implants.

Modern dental implant therapy was pioneered by Brånemark et al. [9], and implant treatment is now considered a crucial part of prosthodontic rehabilitation [10]. Success rates for oral implants reported in studies vary widely, from 51.9% to 95.9% [11–14], depending on the implant system and patient-related factors. As the number of implant procedures rises, complications—including peri-implantitis—have become more frequent [15, 16]. Dental implants often undergo surface modifications, such as acid etching, sandblasting, or plasma spraying, to enhance osseointegration [17, 18]. However, these rough surfaces also create ideal sites for microbial adhesion and biofilm formation, similar to natural teeth. The development of peri-implantitis involves complex multispecies biofilms—most notably containing *P. gingivalis*—on implant surfaces, followed by host immune responses that lead to ongoing alveolar bone loss [19]. Importantly, the microbial species and inflammatory pathways involved in peri-implantitis largely overlap with those seen in periodontal disease, emphasizing shared pathogenic mechanisms across oral infections.

Therefore, strategies focused on preventing and controlling biofilm formation on teeth, restorative materials, and implants are crucial for managing dental caries, periodontal disease, and peri-implantitis. Several approaches have been examined, including surface modifications and the use of antimicrobial agents [20]. However, none have proven long-term effectiveness, and these infections remain significant clinical challenges [21]. With an aging population and increased use of implants in clinical settings, the prevalence of biofilm-related oral diseases is expected to keep rising [22].

In this context, developing durable surface modification strategies capable of suppressing biofilm formation has become an important research priority. Graphene oxide (GO), a two-dimensional nanocarbon material with a high theoretical specific surface area (> 2,000 m²/g) and abundant oxygen-containing functional groups, has attracted interest as a surface modifier and carrier material [23–25]. In dental applications, GO has been investigated for its ability to support the stable incorporation of antibacterial agents and to reduce biofilm formation on restorative and implant materials [26].

Building on these properties, we developed a composite material consisting of GO and cetylpyridinium chloride (CPC), a cationic surfactant widely used as an oral antiseptic, referred to as CPC–GO [27]. In this composite, CPC is associated with the GO substrate, enabling surface-associated antibacterial activity. This study investigates the potential of CPC–GO as a contact-active coating for dental surfaces, including restorative materials and implants, targeting bacterial species associated with dental caries, periodontal disease, and peri-implantitis. Specifically, we evaluated the surface-associated antibacterial performance and coating durability of CPC–GO on representative restorative and implant materials under in vitro conditions.

## Materials and methods

**Fig. 1:**
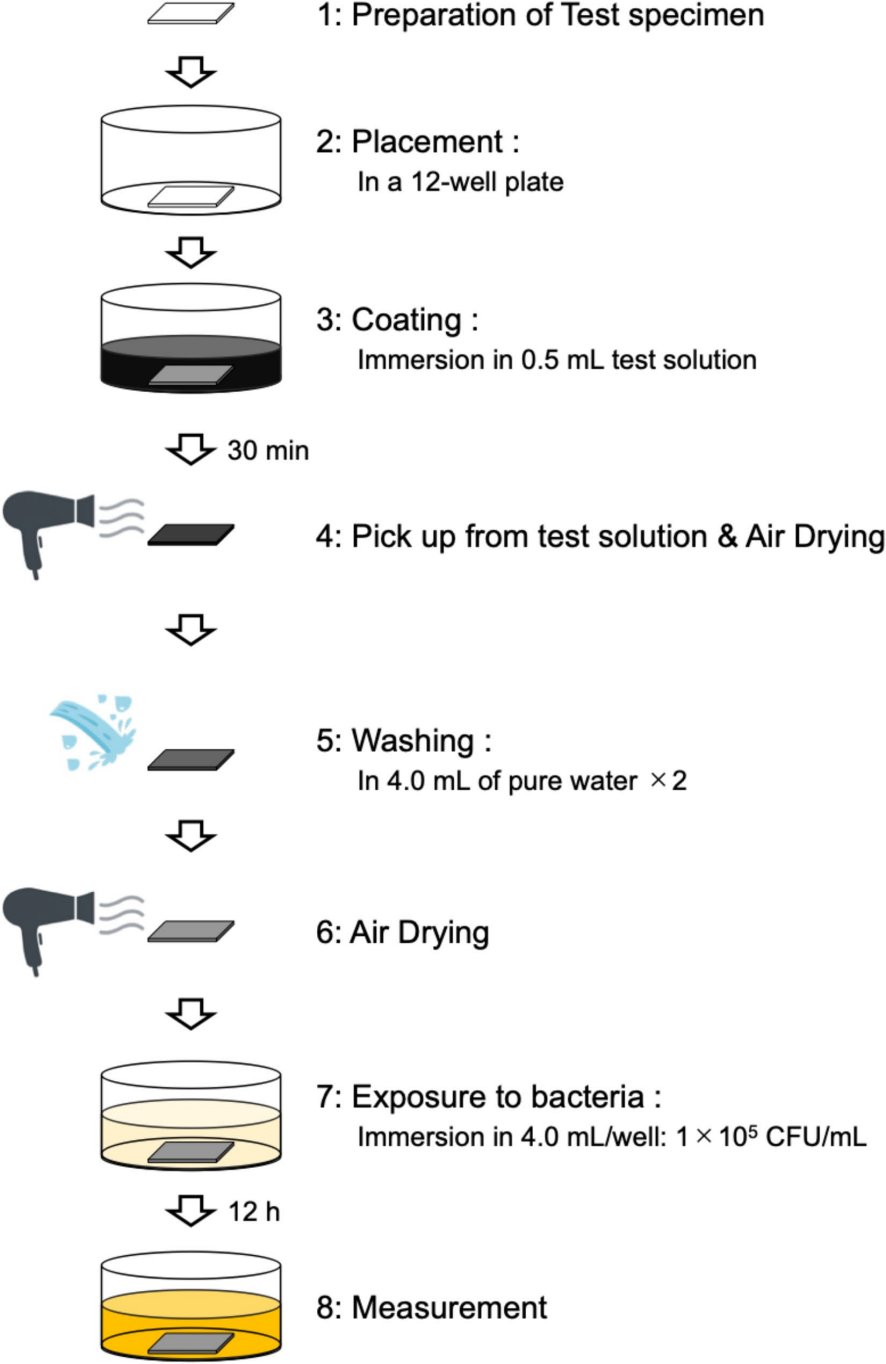
Schematic diagram of preparing CPC–GO composites and applying their coating on dental materials. CPC–GO composites were prepared and applied to composite resin and titanium substrates for 30 min, followed by rinsing and air-drying prior to antibacterial and retention assays

This in vitro experimental study evaluated the antibacterial activity and surface retention of CPC–GO coatings applied to dental restorative and titanium materials. All experimental procedures are outlined as a schematic diagram (Fig. 1). Because the CPC–GO system is intended to function as a surface-bound, contact-active coating rather than as a diffusible antimicrobial agent, evaluation focused on surface-associated antibacterial effects.

### Preparation of GO and CPC-GO

An aqueous dispersion of GO nanosheets was prepared following a method previously developed by our group [28]. The CPC-GO composite was created by reacting GO with CPC (Sigma-Aldrich, St. Louis, MO, USA) to facilitate the adsorption of CPC onto the GO surface. The weight ratio of CPC to GO was 1:1. Specifically, GO suspension was combined with CPC at a final concentration of 1.0 mg/mL under vigorous stirring, then subjected to mild sonication in a bath-type sonicator for 30 min, as previously described [27]. All solutions were prepared using ultrapure water from a Milli-Q IQ 7003 purification system (Merck Millipore, Darmstadt, Germany).

### Preparation of test solutions

The concentrations of GO and CPC–GO prepared by the above method were adjusted to 0.2% (w/v) using 99.5% ethanol (EtOH; Sigma-Aldrich). As control solutions, phosphate-buffered saline (PBS; pH 7.4; Thermo Fisher Scientific, Waltham, MA, USA), CPC (0.2% w/v in EtOH), and EtOH alone were prepared under the same conditions. All solutions were stored at room temperature and used within 24 h after preparation.

### Test specimens and surface treatments

Test specimens were prepared using silicone molds measuring 10 × 10 × 1 mm. For the composite resin (CR) plates, a flowable composite resin (Clearfil Majesty ES Flow High; Kuraray Noritake Dental Inc., Tokyo, Japan) was injected into the mold, covered with a transparent glass plate, and light-cured with a curing unit (PenCure 2000; Morita Corp., Kyoto, Japan) for 10 s on each side. After curing, the CR plates were polished with a silicone polishing point (HP R2; Shofu, Kyoto, Japan) at 10,000 rpm, followed by additional polishing with a finishing wheel (Compo-Master; Shofu) at 10,000 rpm; each surface was polished for 5 min. All specimens were then ultrasonically cleaned in distilled water for 10 min and air-dried. All specimen preparations and surface treatments were performed by the same operator.

For the titanium plates (Ti; Engineering Test Service, Osaka, Japan), commercially available plates of the same dimensions were used without additional polishing.

Each specimen was immersed in 0.5 mL of the respective test solution for 30 min, dried once using a dryer (KHD-W730; Koizumi Seiki, Osaka, Japan), rinsed twice with 4.0 mL of pure water, and then thoroughly dried again using the same dryer.

### Bacteria

*Streptococcus mutans* (*S. mutans*) ATCC 25,175 (ATCC, Manassas, VA, USA) was cultured aerobically in brain heart infusion (BHI) broth (Becton, Dickinson and Company, Franklin Lakes, NJ, USA) at 37 °C for 6 h to reach the logarithmic growth phase. The bacterial suspension was then diluted in fresh BHI medium to 1.0 × 10^5^ CFU/mL. Bacterial turbidity in each test solution was measured using a photometer (Miniphoto 518R; Taitec, Saitama, Japan) at 660 nm [27]. Subsequently, 4 mL of the prepared suspension was added to each specimen, which was then incubated at 37 °C for 12 h.

*P. gingivalis* W83 was cultured in modified GAM broth (Shimadzu Diagnostics, Tokyo, Japan) at 37 °C under anaerobic conditions (oxygen concentration < 0.1%, CO_2_ <15%) using an AnaeroPack™ system (Mitsubishi Gas Chemical Co., Inc., Tokyo, Japan). After reaching the late exponential phase, the culture was adjusted to 1.0 × 10^5^ CFU/mL with fresh medium. The same inoculation volume (4 mL per specimen) and incubation conditions (37 °C for 12 h) were used for assays involving *P. gingivalis*.

### Measurement of the amount of adenosine triphosphate (ATP)

Bacterial viability was assessed by measuring intracellular ATP levels. After 12 h of exposure to the test solutions, ATP content was determined using the Lucifer HS kit along with a Lumi-tester C-110 (Kikkoman Bio-Chemiphar Co., Ltd., Tokyo, Japan), based on the luciferin–luciferase bioluminescence reaction. The luminescence output was expressed as relative light units (RLU), covering a detection range of 1.0 × 10^− 16^ to 3.0 × 10^− 11^ mol of ATP. To remove extracellular ATP before measurement, adenosine phosphate deaminase was added [27, 29, 30].

### Fluorescence-based qualitative analysis of bacterial colonization

Fluorescence-based analysis of bacterial viability was conducted using the LIVE/DEAD^®^ BacLight™ Bacterial Viability Kit (Thermo Fisher Scientific, Waltham, MA, USA). SYTO9 and propidium iodide were mixed at a 1:1 ratio, and 3 µL of the dye solution mixture was added to 1,000 µL of the bacterial suspension, following the manufacturer’s instructions. After staining, the samples were examined with a fluorescence microscope (BZ-X810; Keyence Corporation, Osaka, Japan), and the images obtained were analyzed using the built-in Hybrid Cell Count function. SYTO9-positive cells were identified as live bacteria, while propidium iodide–positive cells were identified as dead bacteria. The viability ratio was determined based on the relative counts of live and dead cells.

### Raman spectroscopy

The surface properties of the CPC-GO-treated samples were analyzed using a laser Raman spectrophotometer (NRS-3100; Japan Spectroscopic Co., Ltd., Tokyo, Japan). The samples were prepared in the same manner as described above. Measurements were taken with an exposure time of 10 s. In addition to the CR and Ti specimens, a GO-coated silicone substrate was used as a reference control for Raman measurements, since silicone (10 × 10 × 1 mm; Sansho, Japan) provides a low-fluorescence background suitable for baseline comparison.

### Microscopic observation

The surfaces of the test specimens were observed using an optical microscope (RX-100, HR2500; Hirox Co., Ltd., Tokyo, Japan), both after drying and washing.

### Statistical analysis

Statistical analysis was conducted using one-way analysis of variance (ANOVA), followed by Tukey’s multiple comparisons test. All analyses were performed with GraphPad Prism, version 8.4.3 (686) (GraphPad Software, San Diego, CA, USA). Statistical significance was set at *P* < 0.05.

## Results

### Antibacterial activity of GO alone

**Fig. 2:**
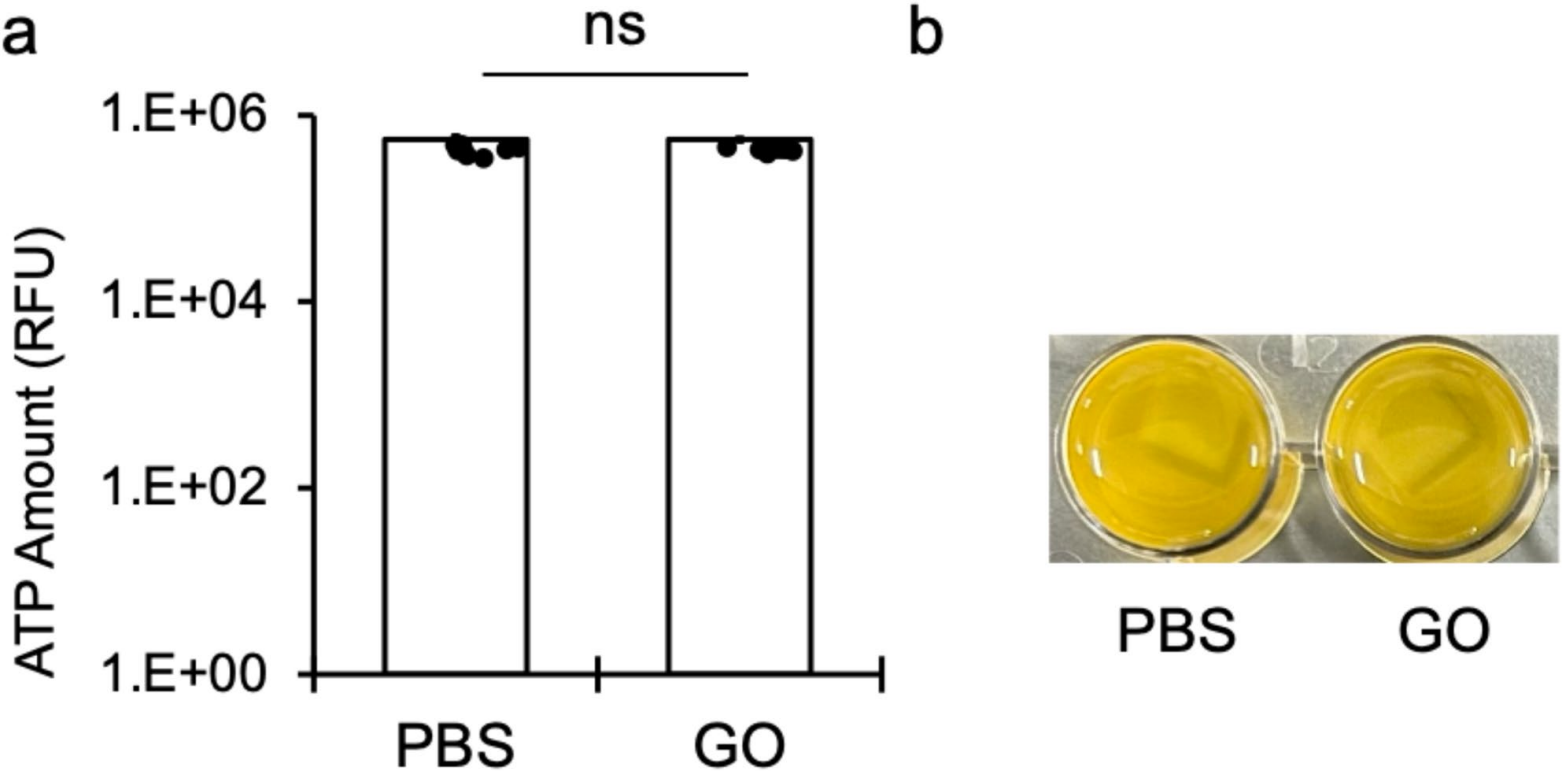
Antibacterial activity of GO alone on CR plates against S. mutans. (**a**) ATP levels after 12 h-incubation on CR surfaces exposed to PBS or GO (mean ± SD of three independent experiments, each measured in triplicate) showed no significant difference. (**b**) Visual appearance of bacterial suspensions showed similar turbidity between PBS- and GO-treated samples, indicating negligible antibacterial effects of GO alone

The ATP assay showed no significant difference in bacterial viability between the PBS and GO groups (Fig. 2a). Both groups had similarly high ATP levels, indicating that GO at the tested concentration did not have measurable antibacterial effects on S. mutans. In line with these quantitative results, the appearance of the bacterial suspensions showed no observable differences between PBS- and GO-treated samples (Fig. 2b). 

### Antibacterial effects of CPC–GO on CR against S. mutans

**Fig. 3:**
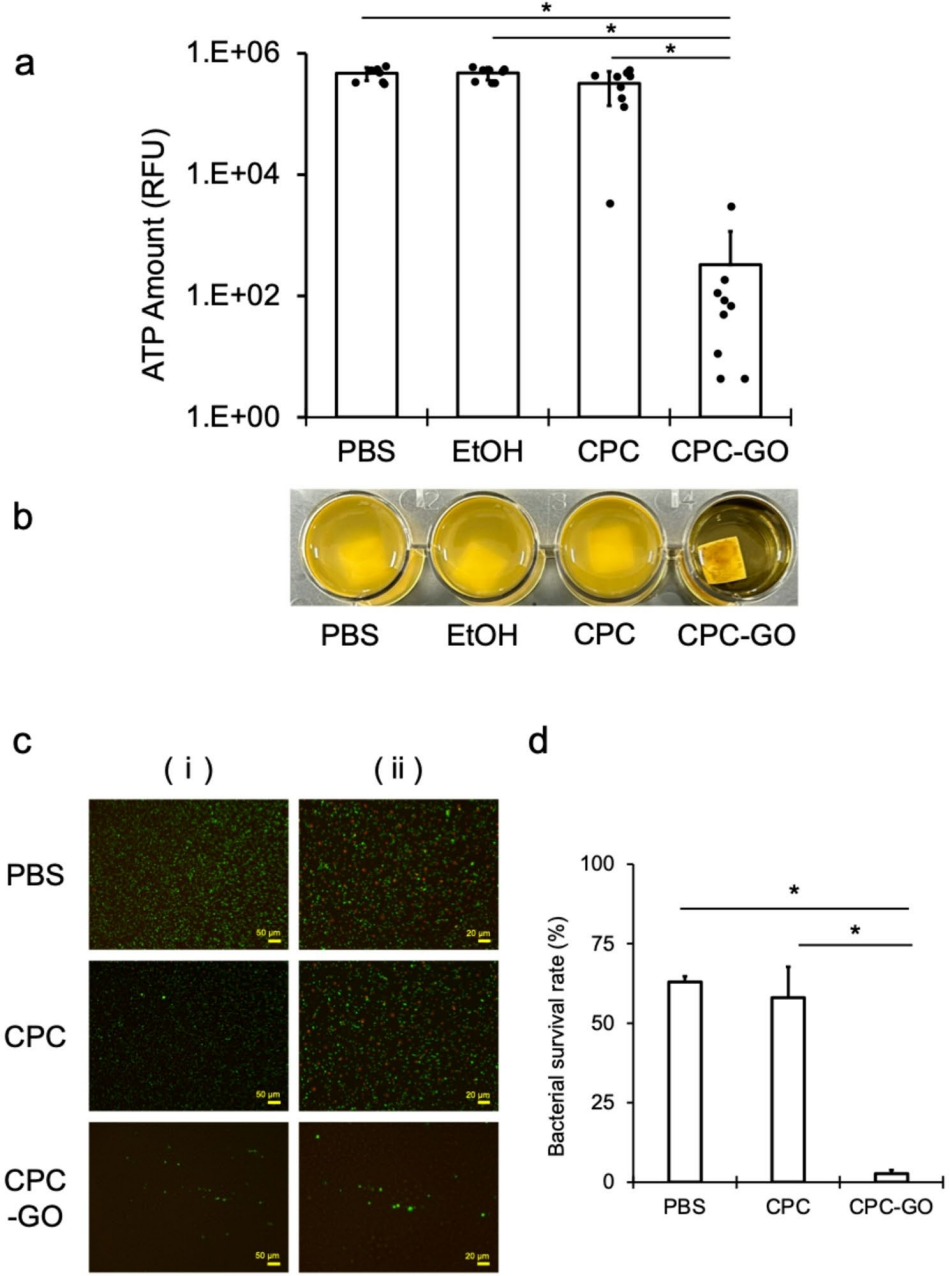
Antibacterial effects of CPC–GO on CR against *S. mutans.* (**a**) ATP levels after 12 h-incubation on CR surfaces treated with PBS, EtOH, CPC, or CPC–GO (CPC concentration: 0.2% w/v; CPC: GO = 1:1 by weight) (mean ± SD of three independent experiments, each measured in triplicate). CPC–GO significantly reduced bacterial ATP compared with all controls (*P *< 0.05). (**b**) Visual appearance of bacterial suspensions after incubation showed increased turbidity in control groups, while CPC–GO kept suspensions clear. (**c**) Representative fluorescence microscopy images of live (green) and dead (red) bacteria, with scale bars of 50 μm for (i) and 20 μm for (ii). (**d**) Quantitative analysis of bacterial viability from fluorescence images (mean ± SD of three independent experiments) shows significantly reduced survival on CPC–GO surfaces

Bacterial exposure to CPC–GO–coated CR surfaces resulted in a significant decrease in ATP levels compared to all negative control groups (PBS, EtOH, and CPC alone), indicating strong antibacterial activity (Fig. 3a). After incubation, bacterial suspensions in all negative controls (PBS, EtOH, and CPC alone) became increasingly turbid, whereas the CPC–GO group showed no visible increase in turbidity (Fig. 3b). These visual observations aligned with the markedly reduced ATP levels seen for CPC–GO.

### Antibacterial effects of CPC–GO on Ti against P. gingivalis

**Fig. 4:**
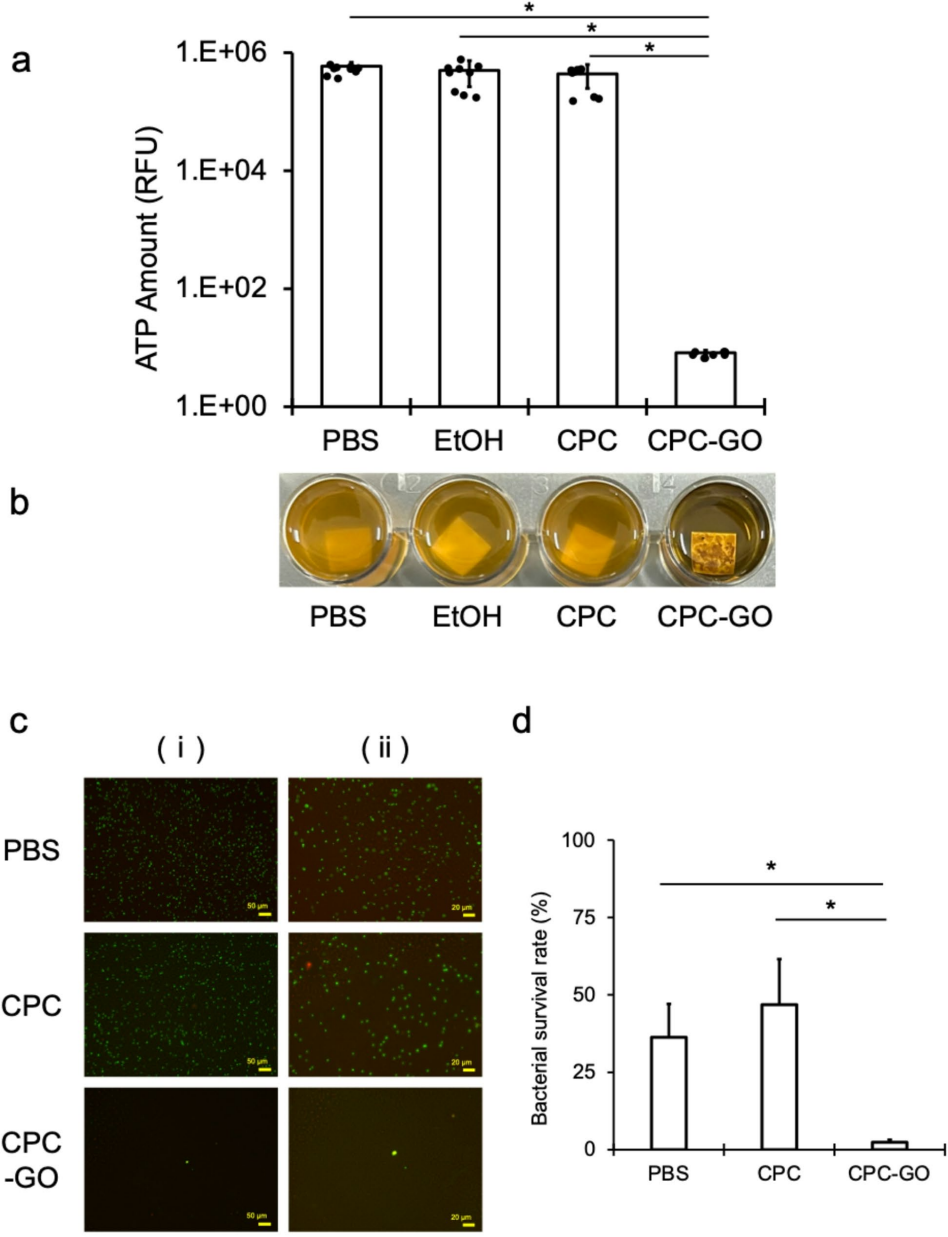
Antibacterial effects of CPC–GO on Ti surfaces against *P. gingivalis. *(**a**) ATP levels after 12 h-exposure on Ti surfaces treated with PBS, EtOH, CPC, or CPC–GO (CPC concentration: 0.2% w/v; CPC: GO = 1:1 by weight) (mean ± SD of three independent experiments, each measured in triplicate). CPC–GO significantly reduced ATP levels compared to all controls (*P *< 0.05). (**b**) Visual assessment revealed that control groups exhibited turbidity typical of P. gingivalis growth, while CPC–GO-treated cultures remained clear. (**c**) Representative fluorescence microscopy images of Ti surfaces showing live (green) and dead (red) bacteria, with scale bars of 50 μm for (i) and 20 μm for (ii). (**d**) Quantitative analysis of bacterial viability from fluorescence images (mean ± SD of three independent experiments) confirmed significant suppression of P. gingivalis on CPC–GO-coated surfaces

Exposure of *P. gingivalis* to CPC–GO–treated Ti surfaces caused a significant decrease in ATP levels compared to all negative control groups (PBS, EtOH, and CPC alone), confirming strong antibacterial activity on the Ti surface (Fig. 4a). During incubation, all control cultures showed increased turbidity typical of *P. gingivalis* growth, while the CPC–GO group showed no visible increase in turbidity (Fig. 4b). These visual results matched the significantly lower ATP levels seen in the CPC–GO group.

Fluorescence microscopy further validated this trend, showing that viable bacterial cells were plentiful on Ti surfaces in the PBS and CPC groups, while their presence was notably reduced on the CPC–GO–coated surface (Fig. 4c and d).

### Retention of GO and CPC-GO on the sample surface

**Fig. 5:**
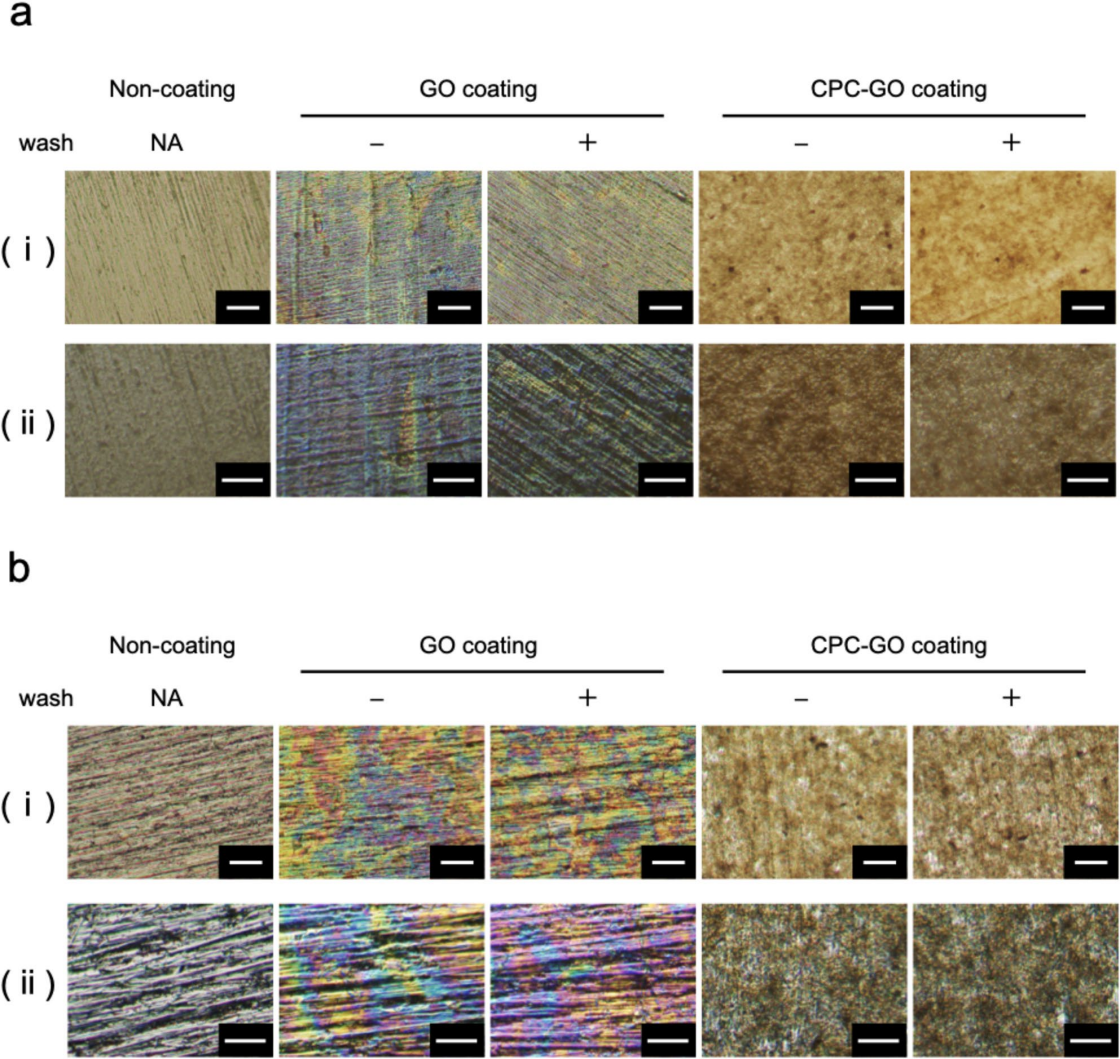
Optical microscopy images of GO and CPC–GO coatings on material surfaces. (**a**): CR, (**b**): Ti. In both materials, GO-associated surface features remained visible after washing, demonstrating stable retention of GO and CPC–GO on the substrate surfaces. Panels labeled with (i) include a 50 μm scale bar, while those labeled with (ii) include a 25 μm scale bar

**Fig. 6:**
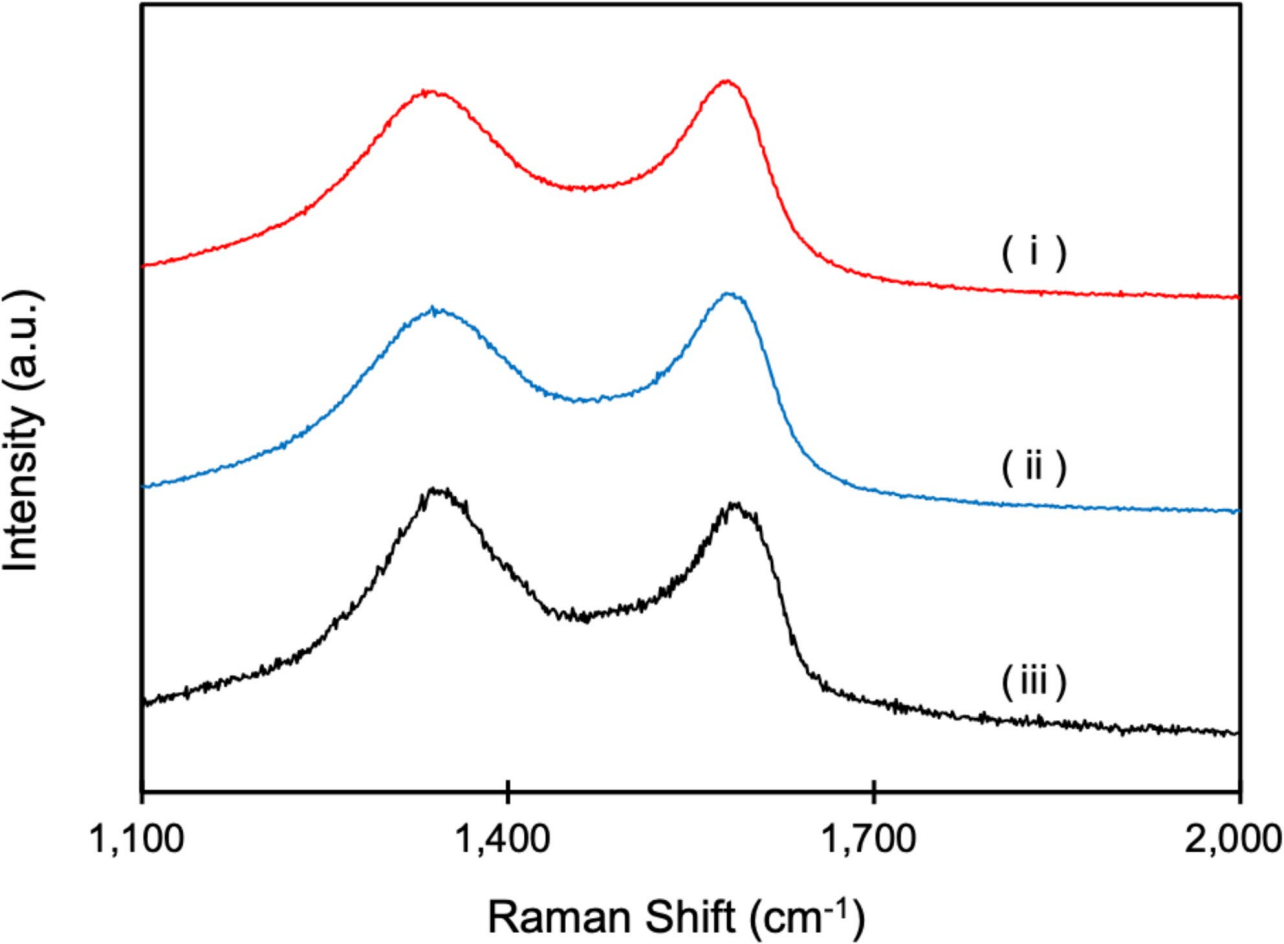
Raman spectral analysis of GO and CPC–GO coatings on CR and Ti surfaces. Raman spectra of material surfaces after treatment with GO or CPC–GO and subsequent washing. (i) CPC–GO on CR, (ii) CPC–GO on Ti, and (iii) GO-coated on silicone substrate used as a reference control. All spectra showed characteristic D (1,360 cm− 1) and G (1,580 cm− 1) bands, confirming the presence of GO. No significant differences in the intensity ratios of D and G (ID/IG) were observed between GO- and CPC–GO–treated samples on either substrate. The ID/IG values for (i), (ii), and (iii) were 0.96, 0.95, and 1.01, respectively

Microscopic observations of both CR (Fig. 5a) and Ti (Fig. 5b) samples showed that GO-derived surface features were visible not only immediately after coating but also after the washing process. In both materials, GO-related textures and optical contrasts remained detectable following washing, indicating that GO sheets stayed on the substrate surface despite rinsing. Additionally, Raman spectroscopy confirmed the presence of the D band (1,360 cm^− 1^) and G band (1,580 cm^− 1^), which are characteristic peaks of GO, in all washed samples (Fig. 6).

## Discussion

Biofilm-related diseases, such as dental caries caused by *S. mutans* and periodontal or peri-implant infections involving *P. gingivalis*, remain significant challenges in dentistry. While primary prevention on tooth and root surfaces is crucial, there is increasing interest in strategies that inhibit microbial adhesion and growth on dental material surfaces to lower the risk of secondary disease. In this study, we examined the beneficial surface properties of GO and explored whether a composite coating of GO and the established antimicrobial agent CPC could offer effective antibacterial activity and durable surface retention against common cariogenic and periodontal pathogens on frequently used dental materials. Specifically, we tested CPC–GO on CR, a resin-based restorative material, and Ti, the main material for dental implants, to evaluate its potential usefulness in diverse dental applications.

In this study, we first checked whether GO alone exhibited antibacterial activity. As shown in Fig. 2, the ATP levels and turbidity of *S. mutans* cultures exposed to GO were comparable to those of the PBS control, indicating that GO had no detectable antibacterial effect under our experimental conditions. This supports our concept in which GO serves as an inert carrier matrix for CPC and confirms that CPC is responsible for the strong antibacterial effects.

Next, we examined the antibacterial effects of CPC–GO coating on various dental materials (CR and Ti) and against different pathogens (*S. mutans* and *P. gingivalis*). As shown in Fig. 3, even after washing and air-drying, ATP levels and bacterial viability of *S. mutans* were significantly reduced, demonstrating that the antibacterial effect remained and even surpassed that of CPC alone after washing. Similarly, experiments with Ti substrates and *P. gingivalis* (Fig. 4) confirmed the same antibacterial trend, indicating that the CPC–GO coating can maintain strong antibacterial activity regardless of the substrate material.

Of particular note, *P. gingivalis* is a key pathogen strongly linked to the onset of peri-implantitis. Its strong inhibition on Ti surfaces, while potentially limiting certain clinical uses, indicates significant potential for controlling peri-implant inflammation. The observation of consistent antibacterial effects on both representative dental materials, despite their different properties, suggests that the mechanism of CPC–GO action does not rely on specific material characteristics. This further indicates that the synergy between localized retention of CPC and the carrier function of GO is responsible for the observed antibacterial activity.

Furthermore, to assess the retention of CPC–GO on material surfaces from an engineering perspective, optical microscopy and Raman spectroscopy were used. Previous studies have shown that CPC exhibits sustained release from GO, modulated by its terminal functional groups [27]. In addition, Miyaji et al.. reported multilayered GO–CPC coatings with sustained antibacterial effects in wet environments [31]. However, their system required layer-by-layer deposition and polymer-assisted assembly, unlike our simpler, one-step, binder-free aqueous process. As shown in Fig. 5, CPC–GO remained on the surfaces even after washing, showing results similar to those of GO alone. This finding is consistent with a report that GO has many oxygen functional groups in its layered carbon structure and adheres to the substrate surface through chemical and physicochemical interactions with the functional groups [32]. Additionally, Raman spectroscopic analysis of CR and Ti surfaces after washing and air-drying (Fig. 6) confirmed the presence of both D and G bands in CPC–GO–treated samples, comparable to GO-treated samples, with no significant difference in the I_D_/I_G_. The D band indicates defects in the carbon structure or oxygen-containing functional groups, while the G band results from the C = C stretching vibrations of the sp^2^ carbon framework, indicating the presence of GO [27]. These results collectively demonstrate that CPC–GO stays on material surfaces even after washing and air-drying.

Taken together, based on these observations, the mechanism underlying the antibacterial activity of CPC–GO appears to be primarily contact-dependent, rather than resulting from detergent-like action of freely dissolved CPC. Optical microscopy and Raman spectroscopy confirmed that CPC–GO remained on the material surfaces even after repeated rinsing and air-drying. This observation suggests that the cationic CPC immobilized on the GO surface interacts directly with bacterial cell membranes, causing membrane disruption upon contact. In addition, CPC associates with GO via ionic interactions between its pyridinium group and oxygen-containing functional groups on GO. Although these ionic bonds are relatively stable and reversible, so a limited and gradual release of CPC from the GO surface cannot be ruled out. Such controlled release may partially contribute to the observed antibacterial effects, although it is unlikely to be the dominant mechanism under the conditions used in this study. Overall, CPC–GO exhibits a dual mechanism, with contact-dependent antibacterial activity as the primary contributor, and potential minor contribution from controlled CPC release, consistent with the sustained antibacterial effects observed after washing.

However, several limitations of this study should be acknowledged. First, it was conducted in vitro, and the observed antibacterial activity and surface retention might differ in the complex environment of the oral cavity, where multi-species biofilms, saliva, and mechanical forces such as brushing are present. Nonetheless, the results obtained under these controlled conditions demonstrate that CPC–GO has strong antibacterial activity and stable surface retention, providing a solid foundation for future in vivo studies and potential clinical applications. Second, only two representative dental materials (CR and Ti) and two bacterial species (*S. mutans* and *P. gingivalis*) were tested. However, these materials and pathogens are highly relevant in dental practice, and further testing on other materials and microbial communities could expand the potential use of CPC–GO. Lastly, the long-term stability, biocompatibility, and clinical durability of CPC–GO coatings, especially their resistance to mechanical stresses such as brushing, were not evaluated in this study. Despite this, the current in vitro findings offer a strong basis for future clinical research.

Furthermore, while the present study focused on assessing the antibacterial efficacy and surface retention of CPC–GO coatings on representative dental materials, a systematic evaluation of the minimum inhibitory concentration (MIC) and detailed dose–response relationships for CPC–GO against oral pathogens will be addressed in subsequent studies. Preparation for these investigations, including optimization of CPC loading, is currently underway. These future studies aim to provide a comprehensive understanding of the effective CPC dosage while maintaining the stability and contact-dependent antibacterial activity demonstrated in this work.

Taken together, these findings suggest that CPC–GO has widespread potential in various disease settings, including cariogenic bacteria and pathogens linked to periodontal and peri-implant issues, and may act as an effective new antibacterial coating for dental materials. From a clinical perspective, CPC–GO utilizes CPC, a compound widely used in oral care products, combined with graphene oxide as a carrier material. This relatively simple coating concept may offer practical advantages for future clinical translation, particularly as a surface treatment applied to existing restorative and implant materials.

## Conclusions

This study demonstrated that CPC–GO composites provide long-lasting antibacterial effects against important oral pathogens, including *S. mutans* and *P. gingivalis*, on common dental materials such as composite resin and titanium. The CPC–GO coating stayed securely on the surfaces even after washing and air-drying, highlighting its potential as a durable antimicrobial surface treatment. These results indicate that CPC–GO is a promising approach for preventing biofilm-related oral diseases such as dental caries, periodontal disease, and peri-implantitis, and lay the groundwork for further development toward clinical use in restorative and implant materials.

## Data Availability

Data will be made available upon reasonable request from the corresponding author (okubok@okayama-u.ac.jp).

## Abbreviations

ANOVA: Analysis of variance
ATP: Adenosine triphosphate
BHI: Brain heart infusion
CFU: Colony-forming units
CPC: Cetylpyridinium chloride
CPC–GO: Cetylpyridinium chloride–graphene oxide composite
CR: Composite resin
EtOH: 99.5 % Ethanol
GO: Graphene oxide
PBS: Phosphate-buffered saline
RLU: Relative light units
SD: Standard deviation
Ti: Titanium

## References

[1] Petersen PE, Bourgeois D, Ogawa H, Estupinan-Day S, Ndiaye C. The global burden of oral diseases and risks to oral health. Bull World Health Organ. 2005;83:661–9. 16211157 PMC2626328

[2] Kassebaum NJ, Smith AGC, Bernabé E, Fleming TD, Reynolds AE, Vos T, Murray CJL, Marcenes W. GBD 2015 Oral Health Collaborators. Global, regional, and national prevalence, incidence, and disability-adjusted life years for oral conditions for 195 countries, 1990–2015: a systematic analysis. J Dent Res. 2017;96:380–7. 10.1177/0022034517693566. 28792274 10.1177/0022034517693566 PMC5912207

[3] Marsh PD. Dental plaque as a biofilm and a microbial community—implications for health and disease. BMC Oral Health. 2006;6(Suppl 1):S14. 10.1186/1472-6831-6-S1-S14. 16934115 10.1186/1472-6831-6-S1-S14 PMC2147593

[4] Kolenbrander PE, Palmer RJ Jr, Rickard AH, Jakubovics NS, Chalmers NI, Diaz PI. Bacterial interactions and successions during plaque development. Periodontol 2000. 2006;42:47–79. 10.1111/j.1600-0757.2006.00187.x. 16930306 10.1111/j.1600-0757.2006.00187.x

[5] Bharathi S, Dhanraj G, Sundramurthy VP, Mohanasundaram S. Comprehensive strategies for overcoming dental biofilms: microbial dynamics and innovative methods. Microb Pathog. 2025;205:107690. 10.1016/j.micpath.2025.107690. 40349996 10.1016/j.micpath.2025.107690

[6] Costerton JW, Stewart PS, Greenberg EP. Bacterial biofilms: a common cause of persistent infections. Science. 1999;284:1318–22. 10.1126/science.284.5418.1318. 10334980 10.1126/science.284.5418.1318

[7] Mombelli A, Décaillet F. The characteristics of biofilms in peri-implant disease. J Clin Periodontol. 2011;38:203–13. 10.1111/j.1600-051X.2010.01666.x. 21323716 10.1111/j.1600-051X.2010.01666.x

[8] Schwarz F, Derks J, Monje A, Wang HL, Peri-implantitis. J Periodontol. 2018;89(Suppl 1):S267–90. 10.1002/JPER.16-0350. 29926957 10.1002/JPER.16-0350

[9] Brånemark PI, Adell R, Breine U, Hansson BO, Lindström J, Ohlsson A. Intra-osseous anchorage of dental prostheses. I. Experimental studies. Scand J Plast Reconstr Surg. 1969;3:81–100. 10.3109/02844316909036699. 4924041 10.3109/02844316909036699

[10] Buser D, Sennerby L, De Bruyn H. Modern implant dentistry based on osseointegration: 50 years of progress, current trends and open questions. Periodontol 2000. 2017;73:7–21. 10.1111/prd.12185. 28000280 10.1111/prd.12185

[11] Ranjan M, Almudarris BA, Almalki SA, Miyajiwala J, Irengbam A, Jadhav MS, Makkad RS. Clinical evaluation of long-term survival and success of implant-supported prostheses. J Pharm Bioallied Sci. 2024;16(Suppl):S2156–8. 10.4103/jpbs.jpbs_101_24. 39346402 10.4103/jpbs.jpbs_101_24 PMC11426634

[12] Deporter D, Pharoah M, Yeh S, Todescan R, Atenafu EG. Performance of titanium alloy sintered porous-surfaced implants supporting mandibular overdentures: a 20-year prospective study. Clin Oral Implants Res. 2014;25:e189–95. 10.1111/clr.12043. 23039057 10.1111/clr.12043

[13] Deporter DA, Kermalli J, Todescan R, Atenafu E. Performance of sintered porous-surfaced press-fit implants after 10 years. Int J Periodontics Restor Dent. 2012;32:563–70. 22754904

[14] Simonis P, Dufour T, Tenenbaum H. Long-term implant survival and success: a 10–16-year follow-up of non-submerged implants. Clin Oral Implants Res. 2010;21:772–7. 10.1111/j.1600-0501.2010.01912.x. 20636731 10.1111/j.1600-0501.2010.01912.x

[15] Mombelli A, Müller N, Cionca N. The epidemiology of peri-implantitis. Clin Oral Implants Res. 2012;23:67–76. 10.1111/j.1600-0501.2012.02541.x. 23062130 10.1111/j.1600-0501.2012.02541.x

[16] Singh P. Understanding peri-implantitis: a strategic review. J Oral Implantol. 2011;37:622–6. 10.1563/AAID-JOI-D-10-00134. 21073300 10.1563/AAID-JOI-D-10-00134

[17] Lin HY, Liu Y, Wismeijer D, Crielaard W, Deng DM. Effect of oral implant surface roughness on bacterial biofilm formation and treatment efficacy. Int J Oral Maxillofac Implants. 2013;28:1226–31. 10.11607/jomi.3099. 24066312 10.11607/jomi.3099

[18] Wennerberg A, Albrektsson T. Effects of titanium surface topography on bone integration: a systematic review. Clin Oral Implants Res. 2009;20:172–84. 10.1111/j.1600-0501.2009.01775.x. 19663964 10.1111/j.1600-0501.2009.01775.x

[19] Persson GR, Renvert S. Cluster of bacteria associated with peri-implantitis. Clin Implant Dent Relat Res. 2014;16:783–93. 10.1111/cid.12156. 23527870 10.1111/cid.12052

[20] Subramani K, Jung RE, Molenberg A, Hämmerle CHF. Biofilm on dental implants: a review of the literature. Int J Oral Maxillofac Implants. 2009;24:616–26. 19885401

[21] Jepsen S, Berglundh T, Genco R, Aass AM, Demirel K, Derks J, Figuero E, Giovannoli JL, Goldstein M, Lambert F, Ortiz-Vigon A, Polyzois I, Salvi GE, Schwarz F, Serino G, Tomasi C, Zitzmann NU. Primary prevention of peri-implantitis: managing peri-implant mucositis. J Clin Periodontol. 2015;42(Suppl):S152–7. 10.1111/jcpe.12369. 25626479 10.1111/jcpe.12369

[22] Derks J, Tomasi C. Peri-implant health and disease: a systematic review of current epidemiology. J Clin Periodontol. 2015;42(Suppl):S158–71. 10.1111/jcpe.12334. 25495683 10.1111/jcpe.12334

[23] Novoselov KS, Geim AK, Morozov SV, Jiang D, Zhang Y, Dubonos SV, Grigorieva IV, Firsov AA. Electric field effect in atomically thin carbon films. Science. 2004;306:666–9. 10.1126/science.1102896. 15499015 10.1126/science.1102896

[24] Nishina Y, Eigler S. Chemical and electrochemical synthesis of graphene oxide – a generalized view. Nanoscale. 2020;12:12731–40. 10.1039/D0NR02187K. 32524106 10.1039/d0nr02164d

[25] Le GTT, Chanlek N, Manyam J, Opaprakasit P, Grisdanurak N, Sreearunothai P. Insight into the ultrasonication of graphene oxide with strong changes in its properties and performance for adsorption applications. Chem Eng J. 2019;373:1212–22. 10.1016/j.cej.2019.05.100.

[26] Arias Z, Nizami MZI, Chen X, Chai X, Xu B, Kuang C, Omori K, Takashiba S. Recent advances in apical periodontitis treatment: a narrative review. Bioengineering. 2023;10:488. 10.3390/bioengineering10040488. 37106675 10.3390/bioengineering10040488 PMC10136087

[27] Fujii R, Okubo K, Takashiba S, Bianco A, Nishina Y. Tailoring the interaction between graphene oxide and antibacterial pyridinium salts by terminal functional groups. Carbon. 2020;160:204–10. 10.1016/j.carbon.2020.01.019.

[28] Morimoto N, Suzuki H, Takeuchi Y, Kawaguchi S, Kunisu M, Bielawski CW, Nishina Y. Real-time, in situ monitoring of the oxidation of graphite: lessons learned. Chem Mater. 2017;29:2150–6. 10.1021/acs.chemmater.6b04807.

[29] Okubo K, Ito T, Okamoto K, Yamamoto I, Mizutani H, Kawata Y, Shiota Y, Ito M, Nakamura S, Tai M, Yamamoto T, Takashiba S. Evaluation of the simulator with automatic irrigation control system designed for countermeasures of internal contamination in dental unit water lines. Heliyon. 2020;6:e04132. 10.1016/j.heliyon.2020.e04132. 32566782 10.1016/j.heliyon.2020.e04132 PMC7298401

[30] Okubo K, Ito T, Shiota Y, Kawata Y, Yamamoto T, Takashiba S. Effectiveness and safety of low-concentrated ozonized water for the reduction of contamination in dental unit water lines. Heliyon. 2019;5:e02306. 10.1016/j.heliyon.2019.e02306. 31463403 10.1016/j.heliyon.2019.e02306 PMC6710486

[31] Miyaji H, Kanemoto Y, Hamamoto A, Shitomi K, Nishida E, Kato A, Sugaya T, Tanaka S, Aikawa N, Kawasaki H, Gohda S, Ono H. Sustained antibacterial coating with graphene oxide ultrathin film combined with cationic surface-active agents in a wet environment. Sci Rep. 2022;12:16721. 10.1038/s41598-022-21081-5. 36257962 10.1038/s41598-022-21205-4 PMC9579177

[32] Shao J-J, Lv W, Xiao D, et al. Self-Assembly of Graphene Oxide at Interfaces. Adv Mater. 2014;26:5586–612. 10.1002/adma.201306052. 24852899 10.1002/adma.201400267

